# Indium-Doped ZnO Thin Films Obtained Using Spray Pyrolysis for Position-Sensitive Photodetection

**DOI:** 10.3390/ma18163744

**Published:** 2025-08-11

**Authors:** Pavlina Bancheva-Koleva, Veselin Zhelev, Plamen Petkov, Tamara Petkova

**Affiliations:** 1 Physics Department, University of Chemical Technology and Metallurgy, 8 Kliment Ohridski Blvd., 1756 Sofia, Bulgaria; p.petkov@uctm.edu; 2 Institute of Electrochemistry and Energy Systems, Bulgarian Academy of Sciences, Akad. G. Bonchev Str. Bl. 10, 1000 Sofia, Bulgariatpetkova@iees.bas.bg (T.P.)

**Keywords:** In-doped ZnO, thin film, spray pyrolysis

## Abstract

The main goal of this study was to investigate the properties of ZnO thin films, including pure films and those doped with indium (up to 8 mol%) that was deposited using a spray pyrolysis technique on glass and silicon substrates in order to prepare the position-sensitive structure, Si-SiO_2_-ZnO:In. To this aim, the present work is focused on investigating the effect of indium concentration on the morphology, structure, and optical properties of the films. X-ray diffraction (XRD) analysis reveals a wurtzite polycrystalline structure. Scanning electron microscopy (SEM) images display a smooth and uniform surface characterized by closely packed nanocrystalline clusters. As the indium concentration rises to 8 mol%, the number of nuclei grows, resulting in uniformly distributed grains across the entire substrate surface. The estimated root mean square (RMS) roughness values for the thin films undoped and doped with 3 mol%, 5 mol%, and 8 mol% of ZnO measured using AFM are 6.13, 9.64, and 13.76 nm, respectively. The increase in indium concentration leads to a slight decrease in film transmittance. The measured LPV photosensitivity of about 44 mV/mm confirms the potential use of these thin films in practical applications.

## 1. Introduction

Today, it is a major challenge for humanity to provide clean and sustainable energy. To this end, it needs to be underlined that solar energy is the cleanest and most unlimited of all renewable and sustainable energy sources. The desire to achieve enhanced performance combined with the concept of a highly position-sensitive photodetector has stimulated interest in new materials that show a large lateral photo response. Due to their significance, zinc oxide (ZnO) thin films can serve as both conductive and transparent materials, making them suitable for use as electrodes in solar cells. Photovoltaic devices require such materials to convert sunlight into electrical energy. ZnO thin films are also employed in various other applications, including active emitters for LEDs and laser diodes, acoustic sensors, high-temperature thermoelectric devices, gas sensors, and more [1]. Additionally, these thin films can be utilized in position-sensitive photodetectors such as laser displacement meters, distance sensors, and optical switches. ZnO thin films offer several advantages, including high transparency in the visible region, light-emitting semiconductor properties, material abundance, and nontoxicity [2]. Most notably, zinc oxide is an n-type semiconductor characterized by a wide band gap, a high free exciton binding energy of 60 meV, and a broad range of resistivity values [3]. Thin ZnO films can be generated using a wide range of techniques including sol–gel, thermal evaporation, chemical vapor deposition (CVD), ultrasonic spray pyrolysis (USP), and magnetron sputtering [4,5,6,7,8,9,10]. The spray pyrolysis process is a low-cost, simple experimental setup that allows the deposition of thin films over a large area.

These films have suitable optical properties for use as transparent conductive films with an appropriate dopant. ZnO is doped with a wide variety of ions. Doping is achieved by replacing Zn^2+^ atoms with atoms of higher valence elements like In^3+^. High conductivity can be achieved in zinc oxide thin films through chemical doping with Al, Ga, In, Cu, and Fe [11,12,13,14,15]. Typically, group III elements (Ga, Al, and In) serve as dopant sources as it was verified that they are effective in achieving relatively good surface roughness and electrical properties [16].

In several studies, emphasis has been placed on doping with transition metals to enhance the thermoelectric properties of ZnO [17]; these included Ni, as an example [18], and Sb-doped materials [19]. There are several reports about the doping effects of Mn and Co in the search for dilute magnetic materials [20]. Based on their optical properties, K, Li, and Ba were chosen as appropriate elements [21,22,23]. There have been fewer studies of pure and In-doped ZnO thin films than other element-doped cases so far. These reports suggest that very small amounts if elements have a significant effect on the growth of films and improve the order in the fabric layer to increase the electrical properties in films, which is also one of our goals. Hafdallah, Ynineb, Aida, and Attaf [24] investigated the influence of indium concentration on the physical properties of ZnO thin films. The main goal of our study is using in-house-developed technology to investigate the deposition of thin films with different concentrations and its application as position-sensitive photodetectors which, to our knowledge, has not yet been presented at this point.

## 2. Experimental Procedure

Glass and silicon substrates were employed for the deposition of both undoped and In-doped zinc oxide thin films using the spray pyrolysis technique. InCl_3_ (Alfa Aesar, Karlsruhe, Germany) and zinc acetate dihydrate (Zn (CH_3_COO)_2_·2H_2_O, 99%, Alfa Aesar, Karlsruhe, Germany) served as the precursors, with a solvent mixture of ethanol and distilled water in a 1:4 volume ratio. The solution was stirred for 30 min, with a few drops of acetic acid added to prevent the precipitation of zinc hydroxide. It was then stirred for an additional 2 h to ensure a homogeneous mixture. The solution was kept at a total concentration of 0.1 M (total metal ion concentration), with In concentrations of 3 mol%, 5 mol%, and 8 mol%.

Prior to the deposition process, the glass substrates were cleaned with isopropanol and double-distilled water, while the silicon substrates were cleaned using HF. All measured samples were 1 cm × 1 cm in size to ensure sample uniformity. The thin films were deposited using a spray pyrolysis system developed by V. Zhelev and P. Petkov, patented under N 4384 U1. One of the advantages inherent in this unit is the capacity to control the nozzle amplitude, which influences the film’s deposition. Argon was used as the carrier gas at a pressure of 1.2 bar. The same experimental conditions were applied for both undoped and In-doped films to assess the impact of the dopant on their structural, morphological, and optical properties.

**Figure 1:**
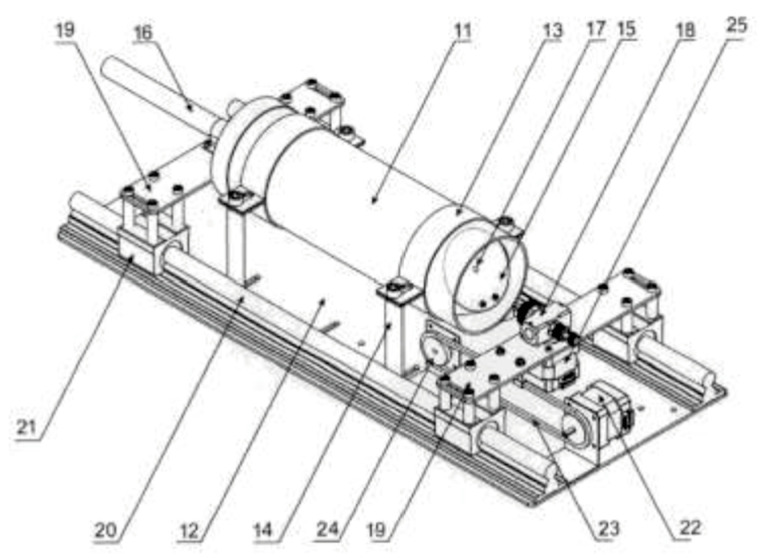
Scheme of spray pyrolysis unit patented under N 4384 U1.

**Figure 2:**
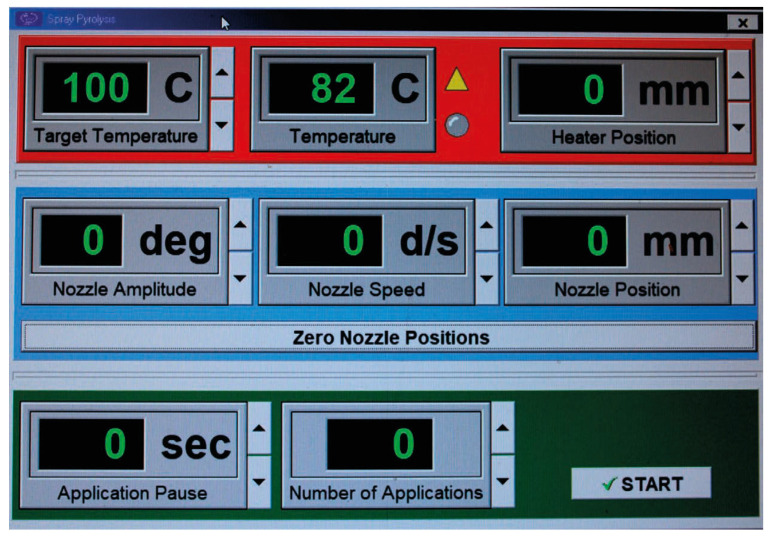
Screen of spray pyrolysis software (version 1.0) with automatable parameters.

A schematic presentation of the spray pyrolysis unit is presented in Figure 1. The thin-film deposition module consists of a tubular quartz chamber (11) with an inner diameter of 100 mm. The tubular quartz chamber (11) is mounted to a metal base (12) using metal rails (13) and brackets (14). Inside the tubular quartz chamber (11), a substrate holder (15) is installed that can be tilted relative to the base (12) and is electrically resistively heated. Using the shaft (16), the substrates placed on the substrate holder (15) are moved in and out of the quartz tube chamber along its axis. A thermocouple (17) is mounted on the front surface of the substrate holder (15) to control the surface temperature. Opposite the substrate holder (15), a mist-type nozzle (18) is installed. It is mounted on a movable support beam (19) capable of linear motion along linear guide shafts (20) via linear bearings (21). The linear bearings (21) are driven by a stepper motor (22) via a chain (23) and shaft (24), and they also allow for controlled rotary motion in the range of 5° to 175°, enabled by a stepper motor (25) mounted on a movable rail directly beneath the nozzle. According to the utility model, the mist-type nozzle (18) from module (4) is connected to a container with a metal salt solution with controlled dosing—working solution module (8)—and to a bottle of compressed inert gas used to generate an aerosol inside the nozzle—module (7). The software of the spray pyrolysis system, presented in Figure 2, allowed for the adjustment of various process parameters, such as target temperature, nozzle amplitude, nozzle-to-substrate distance, nozzle speed, and pressure; these play a significant role in optimizing the deposition process and achieving uniform thin films. The spray cycle (number of applications) was kept constant for all samples. Optimal values were established for the distance between the nozzle and substrate (20–22 cm), spray rate (5 mL/min), nozzle amplitude (45 degrees), deposition temperature (400 °C), and number of applications (40) for both undoped and doped films [25].

The characterization of the structure was conducted using a Philips APD-15 X-ray diffractometer (Eindhoven, The Netherlands), with data collected at ambient temperature within the range of 2θ = 20–85° using Cu-Kα radiation (λ = 1.54178 Å) with the following recording conditions: range of 7–80° 2θ, step size of 0.05° 2θ, and exposure time of 4 s. HighScore Plus version 4.0 and the ICSD (Inorganic Crystal Structure Database) database were used for processing the diffractograms, phase identification, calculation of crystallite size, and microstress and profile analyses. The surface morphology was analyzed using scanning electron microscopy (SEM) (Zeiss, Oberkochen, Germany) with a Zeiss Evo 10, operating in secondary electron mode at an accelerating voltage of 25 keV. Chemical composition was analyzed using an energy dispersive spectroscopy (EDS) detector, Zeiss Smart EDX. Atomic force microscopy (AFM) (Santa Barbara, CA, USA) with an MFP-3D instrument from Asylum Research (Oxford Instruments, Abingdon, UK) was employed to study surface morphology and roughness. The optical properties of the thin films were measured at room temperature using a Shimadzu UV-Vis Spectrophotometer Shimadzu UV-1900i (Kyoto, Japan).

To measure the thickness of the films, we used the Zeta-20 3D Optical profiler (San Jose, CA, USA), which combines a fully integrated microscope with advanced metrology features to provide accurate 3D imaging. Additionally, the F20 thin film analyzer was utilized for precise thickness determination. To prepare the samples, the films on glass substrates were etched with a solution of HCl and distilled water, allowing us to obtain reliable thickness measurements. For the measurement of interface resistance, Hioki RM2611(Ueda city, Japan) (based on the four-point probe method) was used.

## 3. Results and Discussions

### 3.1. Structural Studies

**Figure 3:**
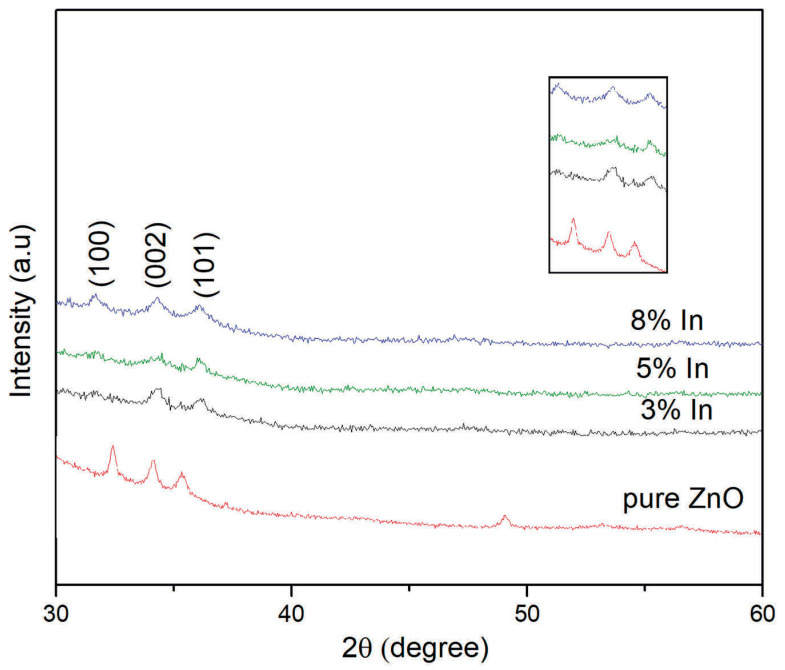
X-ray diffraction patterns of pure and 3 mol%, 5 mol%, and 8 mol% In-doped ZnO thin films.

All the films were found to be polycrystalline with a wurtzite phase and with the c-axis orientation perpendicular to the substrates (Figure 3), as indicated by the XRD measurements. The three prominent peaks corresponding to the (100), (002), and (101) diffraction planes confirm the formation of pure ZnO phases. No peaks related to indium in the XRD patterns indicate that the doped zinc oxide thin films do not contain additional phases, and they also have a hexagonal structure. Upon doping the sample with indium, both intensities corresponding to the (100) and (101) peaks tend to decrease, whereas the intensity of the (002) peak remains nearly unaltered. This trend suggests that the replacement of the Zn atom by the indium atom likely leads to the deformation of the cell, accounting for the changes in the crystallographic characteristics of the film. In the undoped ZnO film, the (100) plane is the most favorable growth orientation. As the dopant concentration increases, a further decrease in the intensities of the (102) and (110) planes is observed. This is attributed to the substrate effect, and it can be explained that the (002) plane has minimum surface energy in the ZnO crystal. The results show that the crystallization quality of the thin film was improved with 8% indium doping, and the (100), (002), and (101) planes were preferred. However, the observed poor crystalline with the addition of the dopant and with the slight increase in its concentration to 5 mol% might be responsible for the decrease in carrier concentration. The indium at a higher concentration resides at the interstitial site and is segregated at the grain boundary, leading to enhanced phonon scattering and ionized impurity scattering, which could also explain the intensity of the peaks with lower and higher concentrations [16]. By nature, all materials are filled with point defects, which gather in grain boundaries and serve as an additional scattering source of charge carriers. All materials inherently contain point defects, which tend to accumulate at grain boundaries and act as an additional scattering source for charge carriers. In most semiconductors, such scattering is currently considered the primary mechanism governing transport as defects can contribute to charge carriers and either scatter or energetically block electron or hole transfer across grain boundaries [26]. It is suggested that the enhanced crystallinity of the ZnO thin film, due to the coalescence of islands achieved by surface and volume diffusion, may be the activation energy for the free electron state occupying the most favorable occupation site of the adsorption atom [27]. Additionally, as calculated using Scherrer’s formula [25,27], the grain size of the ZnO thin film increased from 22 nm to 24 nm at a higher crystallized temperature (600–700 °C). Therefore, a temperature as high as 700 °C promotes ZnO film crystallization. Considering that in this work, crystallization of the indium-doped thin films was carried out at 400 °C, this may justify the disparity in the intensity of the peaks that we obtained compared to other scientists’ achievements [28]. Moreover, the lower processing temperature might be the main reason why the concentration of indium dopants was below the sensitivity of detection.

The profile analysis results are shown in Table 1. The analysis of structural and microstructural features reveals a number of well-established trends. By increasing the amount of indium, the lattice parameter increases slowly. The ionic radii of both ions in fourfold coordination are nearly the same—Zn^2+^ = 0.6 Å and In^3+^ = 0.6 Å—which, geometrically, would not significantly change the dimensions of the lattice.

However, the charge difference may result in oxygen-related defects, possibly in the form of excess oxygen. Also, the effect of the modifier is dependent on the microstructure. The crystallite size is reduced from 25 nm to about 8 nm with respect to the increase in dopant concentration, and this decrease may be due to the possible generation of structural defects. In terms of microstrain, a sudden drop in stress is observed at 3% of the dopant level, and this can be considered as the positive effect induced by a small amount of dopes on structure ordering. However, the stress begins to increase again beyond this concentration.

### 3.2. Morphological Studies

**Figure 4:**
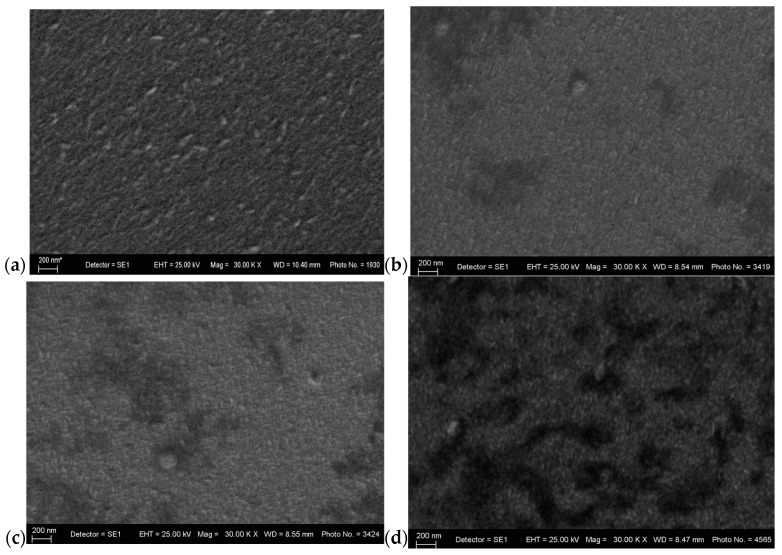
SEM images of (**a**) pure ZnO (* placed by the software), (**b**) 3 mol% In-doped ZnO thin film, (**c**) 5 mol% In-doped ZnO, and (**d**) 8 mol% In-doped thin film.

Figure 4 displays the SEM surface morphology of undoped and In-doped ZnO thin films. The films exhibit a microstructure that comprises several clusters uniformly distributed across the surface. Small dense clusters are seen in the surface morphology of 3 mol% In-doped ZnO thin films. With an increasing In doping concentration, a uniform distribution of particles and a decrease in particle size are also clearly observed. Nuclei grow over all the surface area of the substrate with uniform clusters when the concentration reaches 5 mol%. The crystallite size of pure ZnO thin films is about 60–65 nm. It is observed that In-doped films exhibit a grain size of approximately 35–40 nm, which is smaller than that of pure ZnO thin films, as revealed by the following images. This can be accounted for by the surface migration capacity of the atoms and by the shrinkage of the lattice structure after the substitution of indium. The valence mismatch results in the introduction of one extra electron results in an increased concentration due to a conduction band. Additionally, the difference in ionic radius may yield crystal lattice distortion, leading to internal mechanical stress. Moreover, film morphology depends on the carrier gas, solution flow rate, substrate temperature, precursors and oxidant type and source, and the distance between the nozzle and spray [29]. Grain growth is controlled by the activation energy of self-diffusion, which is the minimum free energy required for atoms or molecules to leave their initial positions and reach a transition state. The energy required for reaction initiation is generally supplied through heating to promote the reaction rate [30,31]. During the solid-phase reaction, a consistently low flow of the carrier gas can decrease the grain size, given their tendency to form aggregates. P. Dhamodharan et al. observed a further reduction from 88 nm to 60 nm in the diameter of In-doped ZnO thin films, and this difference in grain sizes might be due to the lower dopant concentration used [16]. Also, pressure may play an important role in the formation of these films because higher pressures cause faster growth of layers, and this affects grain size. For aluminum-doped thin films, the grain size increases with an increasing temperature, which is attributed to a higher dopant content and the fact that elevated temperatures promote grain growth more easily [32]. Furthermore, major diffraction peaks become weak with an increasing dopant concentration, which is in agreement with the observation that In doping results in reduced crystalline quality. In Figure 2, it appears that the saturated growth of the ZnO (002) orientation along the c-axis is vertically oriented to the substrate. This is consistent with the “drift model”. In this model, “the nucleation of different orientations occurs in the beginning of the film deposition process, and each nucleus competes to grow but only that nuclei will survive which is the fastest in growth on the substrate” [30,33,34]. Comparing the crystallization of ZnO and In-ZnO, it can be seen that a higher concentration of indium caused lattice disorder, which is related to produced stress. E.J. Luna-Arrendondo et al. discuss the morphology of the surface of vacuum-annealed ZnO:In thin films deposited as a function of the film thickness [35].

Indium dopants were incorporated into the 3 mol%, 5 mol%, and 8 mol% In-doped thin films for the EDS spectrum, as shown in Table 2. With an increase in the indium content to 8 at. %, the thin film surface morphology showed lower roughness than other thin films. The EDS data in Table 1 were compared to understand the difference between the particle-like nanophases (In_3_ZnO) and the large reflow zones (In_8_ZnO). The In^3+^ were uniformly dispersed, which led to the contribution of In_2_O_3_ phases being increased. Moreover, a decrease in the mass % and atomic % of Zn and O was observed, which corresponds to the reduction in the size of the formatted clusters.

**Figure 5:**
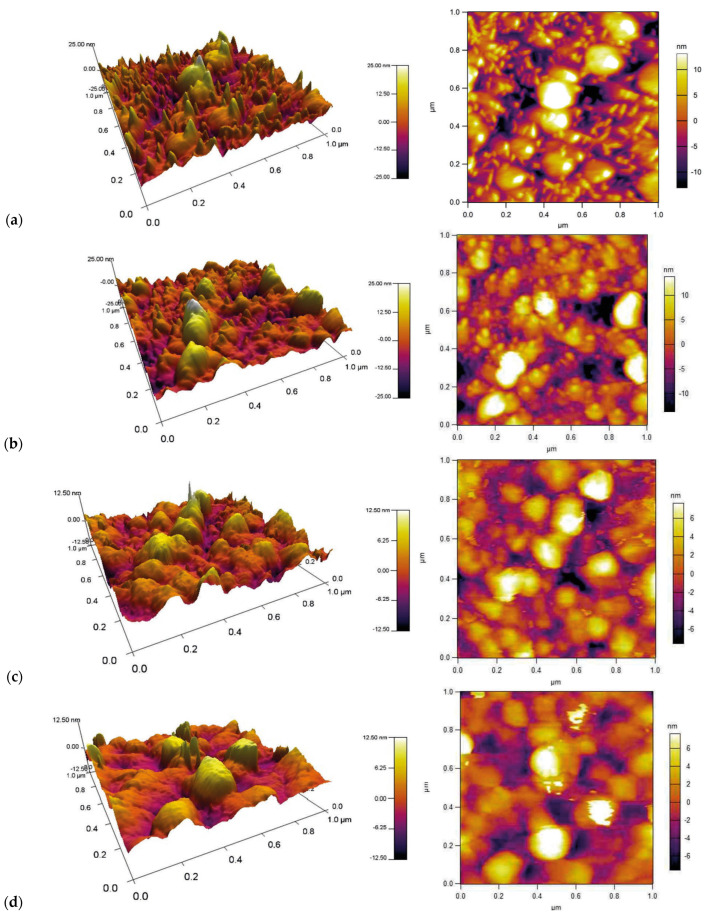
AFM 2D and 3D images of (**a**) pure and (**b**) 3%, (**c**) 5%, and (**d**) 8% In-doped ZnO thin films.

AFM 2D and 3D images of pure ZnO and doped ZnO thin films are represented in Figure 5. The scanning area is 1 × 1 µm; it is seen that these films have a cluster structure, and the clusters are of nanometer sized. The grains’ average size is between 40 and 70 nm derived from 1 × 1 μm area of the film.

The determined root mean square (RMS) roughness of atomic force microscopy (AFM) for undoped and 3 mol%, 5 mol%, and 8 mol% In-doped ZnO thin films are 6.12, 5.82, 2.98, and 2.31, respectively. This study indicates that a comparison of pure and In-doped thin films leads to a decrease in the surface roughness.

### 3.3. Optical Studies

#### 3.3.1. Optical Transmittance

**Figure 6:**
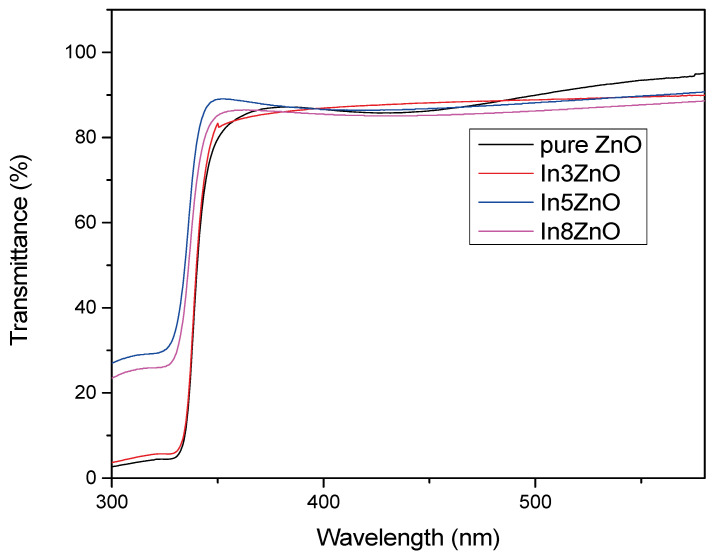
Transmittance spectra of pure and 3 mol%, 5 mol%, and 8 mol% In-doped ZnO thin films.

In this work, the transmission spectra of pure and In-doped ZnO thin films were measured and are presented in Figure 6. The optical transmittance of pure ZnO films in the visible region was lower than that of doped films as indium increases the opacity of oxide thin films. In general, introducing an additive into oxide is expected to reduce its transparency, as observed with the substitution of zinc with indium in the wurtzite structure of zinc oxide. These layers are absorbed in the UV region and first start to transmit around 350 nm at the start of the visible spectrum. Transmission onset, or optical edge, shifts with indium addition. The transmittance of the pure ZnO thin film is 84% and drops by 1% to 83% with the addition of 3% of indium; it further decreases to 82% at an 8% indium content. The longer wavelength shift is due to the addition of 3% indium, whereas the shift to a shorter wavelength is observed when the indium content is 8%. These spectral shifts are associated with changes in the optical band gap, which tends to vary accordingly. Table 3 shows the film thickness data obtained from optical profilometer measurement.

#### 3.3.2. Films’ Thickness

The measured thickness and band gap values of pure and indium-doped ZnO thin films are presented in Table 3. Upon introducing the dopant, the measured films’ thickness increases noticeably and continues to increase slightly with higher indium concentrations. In general, we can conclude that we managed to deposit thin films with a thickness between 750 and 800 nm using the same parameters and only changing the concentration of the dopant.

#### 3.3.3. Band Gap Analysis

The optical band gap is calculated by applying the Tauc model procedure with an accuracy of ±0.002 eV [25]:

αhν = B (hν − E_g_)^n^

where the incident photon energy is hν and the optical band gap is E_g_, B is the constant and, the n value can be 1/2, 3/2, 2, or 3.

The type of inter-band transition—such as direct allowed, direct forbidden, indirect allowed, and indirect forbidden transition—affects the value of n. We used Tauc’s plot by plotting αhν = f(hν) to be able to calculate the band gaps of the films, extrapolating the linear portion of the absorption edge [2,36,37]. The result shows that the Eg value increases from 3.341 to 3.651 eV with the introduction of the dopant, which could be due to the new energy states between the valence and conduction bands. Additionally, the presence of defects may act as recombination centers. The increase in carrier concentration due to the substitution of Zn^2+^ by In^3+^ ions and interstitial indium atoms raises the Fermi level to the conduction band, which leads to widening of the optical band gap [38]. The slight increase in the band gap with an increasing dopant concentration is due to the Burstein–Moss effect. In this case, it is worth pointing out that the optical band gap results indicate that the Burstein–Moss effect is quite strong. Moreover, this occurs in degenerate semiconductors having high carrier concentrations. P. Dhamodharan et al. observed similar behavior when fabricating In-doped ZnO thin films by spray pyrolysis as photoanode in DSSCs [16]. Gupta et al. also reported an increase in the optical band gap in the doped ZnO films and stated that this may be attributed to the increase in carrier concentration on doping [39]. In general, group III elements contribute an additional free electron, resulting in increased n-type conductivity and a concurrent increase in the optical band gap.

#### 3.3.4. Raman Analysis

**Figure 7:**
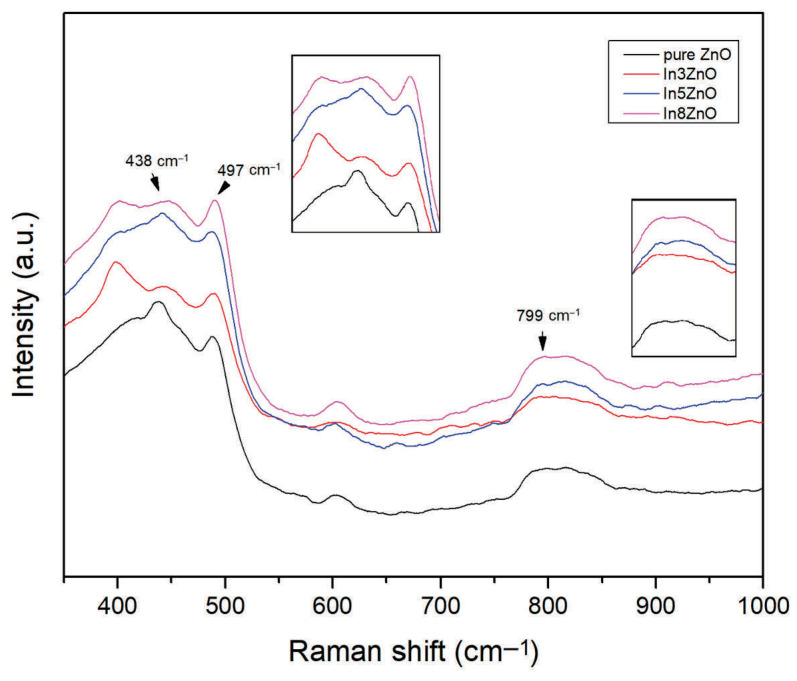
Raman spectra of pure and In-doped ZnO thin films.

The crystalline quality of the films was examined using a Raman spectrophotometer. The Raman spectra of pure and In-doped ZnO thin films deposited on glass substrate are presented in Figure 7. The Raman spectra of the In-doped ZnO thin films show peaks such as those for the wurtzite structure of ZnO, and the dominant E2 (high) mode is localized at around 438 cm^−1^. The addition of indium to the ZnO lattice can shift Raman peak positions and indicate changes in the crystal structure and phonon modes [40]. A maximum peak position was observed at approximately 497–498 cm^−1^ in E2 (high) mode, which is also characteristic of the wurtzite structure of ZnO. The splitting of the strong peak at 438 cm^−1^ with the addition of the dopant reveals the existence of structural defects, impurities, and free carriers. The Raman results are consistent with the XRD data. In general, the sharp mode and the suppressed mode describe films with relatively good crystalline quality [40]. However, a decrease in crystalline quality is observed with an increasing indium concentration. These findings further confirm the structural observations made in the XRD analysis.

## 4. Electrical Properties and Applications

**Figure 8:**
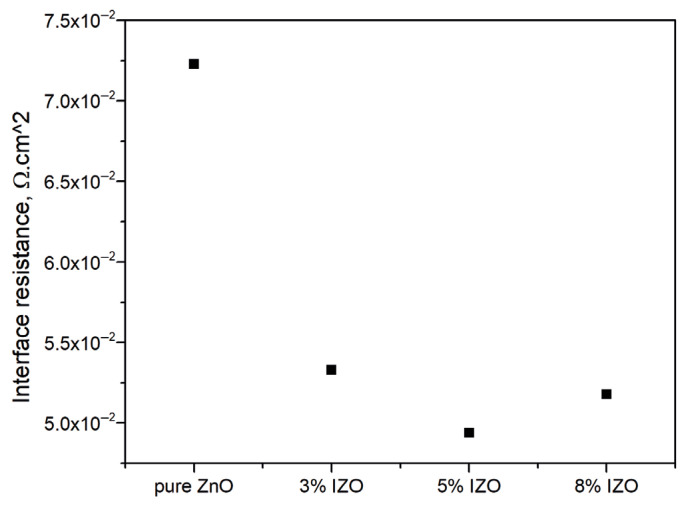
Interface resistance of undoped and In-doped ZnO thin films.

The interface resistance was determined using the Haioki RM2611 electrode resistance technique. Figure 8 shows the sheet resistance of undoped and In-doped thin films. The addition of the dopant (In^3+^) leads to a significant drop in sheet resistance in ZnO thin films. This mainly results from substituting In^3+^ ions to the Zn^2+^ sites in the ZnO lattice, which introduces more free electrons and increases carrier concentration. As the dopant content increases, a further decrease in resistance is observed. This may arise from enhanced electron donation by In^3+^ ions and the generation of oxygen vacancies and interstitial zinc atoms serving as shallow donors that can further enhance conductivity. Additionally, substitutional doping introduces a lattice distortion at the local level that may have led to an improvement in charge mobility by grain boundary modification or deformation and a reduction in scattering centers due to defects. Notably, the lowest sheet resistance appears to be obtained with 5 mol% In doping.

One of the main tasks of this study is to investigate the lateral photo effect (LPV) on position-sensitive structures obtained by depositing In-doped ZnO thin layers on a single-crystal silicon substrate with an oxidized surface. To this aim, these films should possess high optical transmittance and low interface resistance.

**Figure 9:**
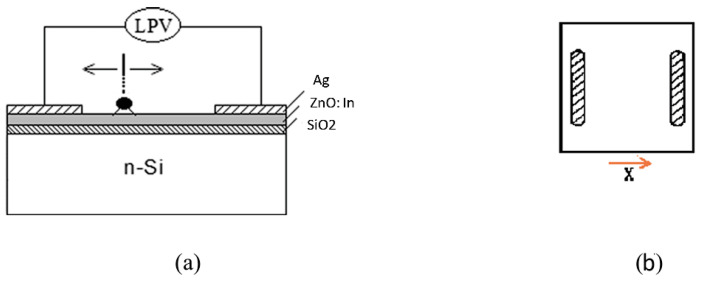
A position-sensitive structure of the Si-SiO_2_-ZnO:In type: (**a**) cross section and (**b**) top view [41].

To prepare the specimens for the study, a rectangular plate of single-crystal Si with an oxidized surface was cut, on which an In-doped layer of ZnO was deposited. Two opposing strip electrodes of Ag paste were prepared on the surface of the metal oxide layer at 7 mm. The appearance of the structure’s cross section and top view are shown in Figure 9.

**Figure 10:**
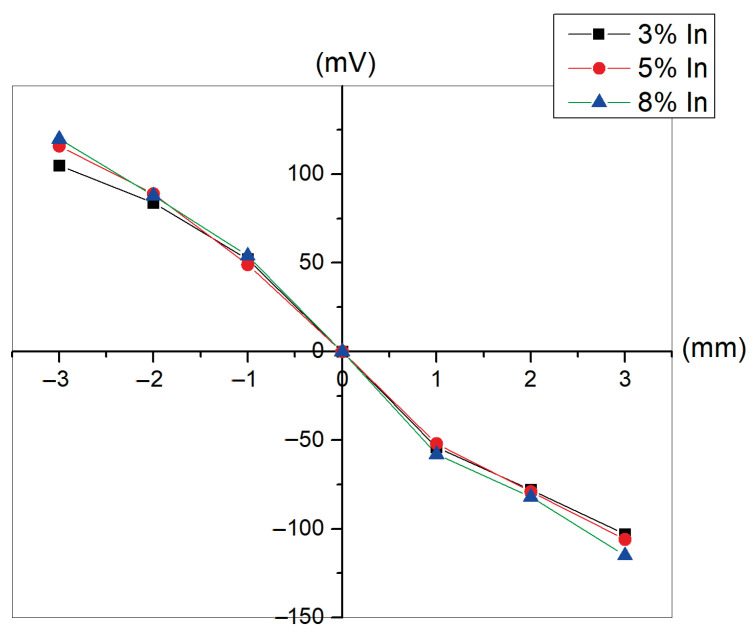
Lateral photovoltage versus laser spot position for ZnO layers doped with 3%, 5%, and 8% indium.

Figure 10 presents the lateral photovoltage as a function of laser spot position for ZnO doped with indium. A good lateral photo effect is characterized by highlight sensitivity, i.e., high photo potential, and very good linearity near the electrodes. The LPV photosensitivity obtained in this study is approximately 44 mV/mm, demonstrating the strong potential of the fabricated samples for practical applications. By contrast, earlier studies carried out by some of the co-authors reported lower LPV sensitivity of around 17 mV/mm, where the lateral photo effect was investigated in a two-dimensional position-sensitive Si–SiO_2_–In_2_O_3_:As structure [42]. This emphasizes the improved LPV performance achieved in the current work, considering the differences in the structures examined. The LPV linearly decreases with the light spot movement over the metal oxide plane in the direction toward the opposite contact, reaching zero at the middle position (x = 0). At this stage, the photovoltage switches its sign and increases in absolute value while the spot moves closer to the electrodes. With further transfer of the light spot toward the other contact, the sign of the LPV reverses. This “sign switch” is related to the reversal in the direction of the net carrier diffusion with respect to the reference electrode. The photovoltage polarity reflects the direction of photogenerated electron (or hole) flow. When the illumination spot is on one side, electrons diffuse toward a specific contact; placing the spot on the opposite side reverses both the diffusion direction and the resulting voltage polarity. The local rise in carrier concentration around the illuminated region generates a diffusion current owing to the gradient in concentration. This results in an observable potential difference between contacts, especially in doped metal oxides where minority carrier diffusion lengths are relatively long and carrier mobility is high. Carrier lifetime is another critical factor as it determines how far photogenerated carriers move before recombination. Due to the longer carrier lifetime, such long diffusion length is supposed to smooth and mirror the LPV response on plane. All samples showed symmetric curves, as shown in Figure 9. As the layer thickness and the dopant concentration increase, a decrease in the positional light sensitivity is observed, also considering the alignment of the crystal grains of the microstructure, which in turn causes light spot scattering and light energy loss. The results presented in this article confirm the potential possibility to create a position-sensitive sensor of the Si-SiO_2_-ZnO:In type.

## 5. Conclusions

In this work, the influence of indium on the structure, optical properties, and morphology of ZnO thin films prepared on glass and silicon substrates was studied. XRD patterns show that polycrystalline films have a wurtzite structure. Doped films seem to change their crystalline structure, as evidenced by the variation in the intensity of the diffraction peaks. The microstructure of the films is observed through SEM analysis, which shows uniform dense clusters throughout the surface. When indium is added as a dopant and the concentration of indium increases, the surface roughness of the film decreases, as evidenced by the changes observed in the AFM studies. The results of UV-VIS spectroscopy show a slight decrease in the transmittance in the visible region for indium-doped ZnO thin films. The sheet resistance was reduced to 32%, the optical bandgap increased from 3.341 eV to 3.651 eV, and the LPV attained a value of 44 mV/mm. The results presented in this article confirm that Si/SiO_2_/ZnO:In structures show LPV material features with the prospects of sensing applications. To gain deeper insights into the underlying mechanisms, it is possible to study the effect of annealing temperature on LPV response in future studies.

## Figures and Tables

**Table 1:** Results from the profile analysis.

In, %	a, Å	c, Å	Size, nm	Strain, %
ICSD	3.2530	5.2070	-	-
0	3.247 (2)	5.216 (4)	25	0.33
3	3.244 (4)	5.197 (7)	14	0.064
5	3.254 (5)	5.211 (8)	9	0.11
8	2.263 (9)	5.22 (1)	8	0.1

**Table 2:** EDS of I_x_ZnO thin films.

Element	Mass (%)			at. %		
	Zn	O	In	Zn	O	In
In_3_ZnO	3.57	7.17	0.09	1.32	10.84	0.02
In_5_ZnO	3.26	6.84	0.09	1.20	10.35	0.02
In_8_ZnO	2.78	6.03	0.10	1.01	8.98	0.02

**Table 3:** The influence of the In content on optical properties.

	Measured Film Thickness, nm	Eg, eV
pure ZnO	350	3.341
In_3_ZnO	748	3.651
In_5_ZnO	760	3.656
In_8_ZnO	800	3.663

## Data Availability

The original contributions presented in this study are included in the article. Further inquiries can be directed to the corresponding author.
